# SARS-CoV-2 and neurodegenerative diseases: what we know and what we don’t

**DOI:** 10.1007/s00702-022-02500-w

**Published:** 2022-04-17

**Authors:** Paul Lingor, Antonia F. Demleitner, Andreas W. Wolff, Emily Feneberg

**Affiliations:** 1 Department of Neurology, School of Medicine, Klinikum rechts der Isar der Technischen Universität München, Ismaninger Straße 22, 81675 Munich, Germany; 2 German Center for Neurodegenerative Diseases (DZNE), Munich, Germany; 3 Munich Cluster for Systems Neurology (SyNergy), Munich, Germany

**Keywords:** COVID-19, Neurodegeneration, Parkinson’s disease, Neurological symptoms, Alzheimer’s disease, SARS-CoV-2

## Abstract

Infection of the CNS with the SARS-CoV-2 can occur via different routes and results in para- or post-infectious manifestations with a variety of neurological symptoms. In patients with neurodegenerative diseases, SARS-CoV-2 is often associated with a higher fatality rate, which is a relevant problem in increasingly older populations. Apart from the direct consequences of an infection in patients with neurodegenerative diseases, indirect consequences of the pandemic such as limited access to care facilities and treatment have negative effects on the course of these chronic disorders. The occurrence of long-lasting neurological symptoms after infection with SARS-CoV-2 indicates a prolonged impact on the CNS. However, while it is known that SARS-CoV-2 affects neuronal populations that are relevant in the pathogenesis of neurodegenerative diseases, it is yet unclear whether an infection with SARS-CoV-2 is sufficient to trigger neurodegeneration. Reflecting on the impact of SARS-CoV-2 on neurodegeneration, we provide a concise overview on the current knowledge of SARS-CoV-2-induced pathology in the CNS and discuss yet open questions in the field.

## Introduction

At the time of writing, almost 500 million people worldwide have been diagnosed with coronavirus disease 2019 (COVID-19), caused by the novel severe acute respiratory syndrome coronavirus-2 (SARS-CoV-2) (WHO 2022). Early in the pandemic, it became clear that SARS-CoV-2 infections harbor the risk of developing neurological manifestations (Ababneh et al. 2020; Mao et al. 2020). It has also become evident that SARS-CoV-2 infections have a negative effect on the outcome of patients with neurodegenerative diseases, predominantly studied in common neurodegenerative diseases such as Alzheimer’s disease (AD) or Parkinson’s disease (PD) (Hu et al. 2021, 2022). In PD, an increased mortality rate was associated with SARS-CoV-2 infection, and an increased hospitalization and mortality rate has been shown for patients with AD (McAlpine et al. 2021; Zhang et al. 2020, 2021a). Data on the clinical outcome of SARS-CoV-2 infections on patients with other neurodegenerative diseases such as amyotrophic lateral sclerosis (ALS), frontotemporal dementia (FTD), or Huntington Disease (HD) are yet limited and will require further studies in the course of the pandemic (De Marchi et al. 2021; Musson et al. 2022). Also, chronic neurological diseases such as multiple sclerosis with increased disability and a progressive course of disease have been associated with higher mortality rates (Prosperini et al. 2022; Barzegar et al. 2021).

So far, different mechanisms have been considered as a cause of neurological symptoms or acceleration of existing neurological conditions during SARS-CoV-2 infection. These include direct effects of the virus on the central nervous system (CNS) by, e.g., entering the brain via nasal or oral routes and infection of neuronal populations (Meinhardt et al. 2021) and para- or post-infectious effects such as triggering inflammation and auto-immune reactions (Zubair et al. 2020; Franke et al. 2021). Those effects of SARS-CoV-2 on the CNS have potential implications for the development of long-term neurological conditions including neurodegeneration. In addition, systemic effects during severe sepsis can trigger neurological symptoms (Heneka et al. 2020; Widmann and Heneka 2014), and depend on the outcome and management of the acute disease.

In this review article, we will summarize recent evidence of effects of SARS-CoV-2 infection on the CNS and its implications for neurodegenerative diseases. We identified the existing evidence through author knowledge and PubMed searches from database inception, to March, 2022 using the search terms: “neurofilament”, “neurodegeneration”, “neurodegenerative diseases”, “Parkinson disease”, “Alzheimer disease”, “amyotrophic lateral sclerosis”, “dementia”, “multiple sclerosis”, “Covid-19”, and “SARS-CoV-2”.

### Nervous system involvement in SARS-CoV-2-infection

When COVID-19 emerged, it became evident that the involved pathogen, the novel SARS-CoV-2 variant, was more transmittable between humans than its predecessor SARS-CoV-1 (Liu et al. 2020b). Similar to its predecessor, SARS-CoV-2 uses the same densely glycosylated spike (S) protein on the viral surface to bind to the peptidase domain of the angiotensin-converting enzyme 2 receptor (ACE2) on mammalian host cells to enter cells yet with much higher affinity (Wrapp et al. 2020). ACE2 is an extracellular enzyme with a transmembrane domain located toward the cell surface. Upon protease activation, soluble ACE2 is released into the extracellular space, where it physiologically processes angiotensin peptides as part of the angiotensin–renin pathway, while the transmembrane domain of ACE2 is internalized into the cell. During the internalization process, SARS-CoV-2 bound to ACE2 is either taken up by clathrin-coated vesicles or directly through involvement of the ACE2-specific transmembrane protease serine subtype 2 (TMPRSS2) (Wu et al. 2021). ACE2 is predominantly expressed in airway epithelia and lung parenchyma, kidney cells, small intestine, and vascular endothelia throughout the body, but also widely throughout the CNS (Deinhardt-Emmer et al. 2021). Predilection sites of ACE2 in the brain are the posterior cingulate gyrus, motor cortex, olfactory bulb, middle temporal gyrus, substantia nigra, and the ventricles, where it is expressed in neurons, astrocytes, and oligodendrocytes (Xia and Lazartigues 2008; Chen et al. 2020).

Loss of smell and taste were among the first CNS-related symptoms associated with SARS-CoV-2 infection, providing the early support for the hypothesis of direct entry of SARS-CoV-2 into the brain via the olfactory and oral mucosa. Although loss of smell is a common symptom in COVID-19 patients, its manifestation likely depends on the genetic variability of the virus and its host such as differences in ACE2 and TMPRSS2 expression (Li et al. 2020; Korber et al. 2020; Shang et al. 2020; von Bartheld et al. 2020; Strafella et al. 2020; Williams et al. 2020). Studies with SARS-CoV-infected mice showed that virus antigen is found in the olfactory bulb and subsequently in connected brain regions such as the piriform cortex, basal ganglia, midbrain, and cardiorespiratory region of the medulla (Netland et al. 2008). The detection of SARS-CoV-2 in human brain tissue has been challenging and likely depends on the severity of the CNS involvement. Whereas no evidence of SARS-CoV-2 was found in the brain of 19 infected patients without neurological symptoms (Hirschbuhl et al. 2021), a post-mortem study of 33 SARS-CoV-2 patients with neurological symptoms found viral RNA in the nasal mucosa and to a lesser extend in the ophthalmic and oral mucosa as well as in the medulla oblongata (Meinhardt et al. 2021). This study suggests that infection of the CNS by SARS-CoV-2 in patients with neurological symptoms is mediated by transsynaptic transmission across peripheral olfactory neurons to connected brain areas. Although this study did not detect SARS-CoV-2 in the carotid blood vessels and thus argued against a systemic infection via the blood stream, this route of infection cannot be completely ruled out. In a more detailed study of the olfactory mucosa, the virus was found in sustentacular and stem cells of the mucosa while absent from olfactory receptor neurons (ORN) (Butowt and von Bartheld 2020).

Alternative routes of CNS infection have been described across the blood–brain barrier (BBB), ACE2 expressing vascular endothelial cells, or indirect trafficking of the virus via blood leukocytes under increased permeability of the BBB (Zubair et al. 2020; Gomes et al. 2021). By infecting inflammatory cells such as monocytes, macrophages, dendritic cells, and lymphocytes, the virus is able to initiate a cascade of cytokine release and an overwhelming activation of leucocytes which is responsible for subsequent cellular damage. Alternative explanations for SARS-CoV-2 CNS pathogenicity come from its association with auto-immune diseases (Tang et al. 2021). Its complex transcriptome, its ability to mimic human proteins, its ability to promote the generation of autoantibodies, and activate cytokine responses trigger auto-immune responses (Kim et al. 2020; Kanduc 2021; Zhou et al. 2020c). Neuroimmune manifestations such as Guillain–Barré syndrome (GBS), myasthenia gravis, acute disseminated encephalomyelitis (ADEM), or encephalitis suggest an autoantibody-mediated inflammatory response in certain neuronal populations (Toscano et al. 2020; Parsons et al. 2020; Zhou et al. 2020b; Muhammed et al. 2021; Restivo et al. 2020; Foresti et al. 2021). In blood and CSF of SARS-CoV-2-infected patients with neurological symptoms, known autoantibodies against the NMDA-receptor or intracellular Yo-antigen were found, but also antibody binding on yet undetermined antigen epitopes on vessel endothelium, astrocytic proteins, neuropil of basal ganglia, hippocampus, or olfactory bulb from such samples was discovered (Watson et al. 2021; Franke et al. 2021; Mulder et al. 2021). This implies immunosuppressive therapy or plasmapheresis as a possible treatment for autoantibody-associated neurological symptoms.

### SARS-CoV-2-triggered neurological symptoms and complications

Neurological symptoms have been described during and post-infection with SARS-CoV-2 (Rifino et al. 2021; Ellul et al. 2020). The clinical presentation of neurological and encephalopathic findings ranged from mild headache and confusion over fluctuating levels of consciousness and extrapyramidal movement disorders (e.g., myoclonus) to seizures and coma (Garg et al. 2021; Romero-Sanchez et al. 2020). Although the number of neurological complications might be lower in SARS-CoV-2 compared to other viruses, the scale of the pandemic renders the overall number of neurological complications and diseases a relevant socioeconomic burden (Ellul et al. 2020). As direct evidence of viral DNA cannot be measured in the CNS of living patients, cerebrospinal fluid is used as an alternative source. However, only two case reports reported positive results for SARS-CoV-2 DNA in the CSF of COVID-19 patients with associated encephalitis, while larger retrospective studies of COVID-19 patients with neurological diseases had negative CSF measurements (Moriguchi et al. 2020; Zhou et al. 2020a; Destras et al. 2020; Neumann et al. 2020; Bellon et al. 2021). Nevertheless, several case studies confirmed neurological diseases associated with SARS-CoV-2 infection. Mild encephalopathies with transient symptoms such as altered consciousness (19.1% of 841 patients) or bradypsychia and disorientation (10.1% of 841 patients) were found to occur rather commonly often accompanied by findings of unspecific T2/FLAIR hyperintensity (35% of 20 patients) and ischemic infarcts (31% of 108 patients) in neuroimaging (Romero-Sanchez et al. 2020; Mahammedi et al. 2020; Radmanesh et al. 2020). Severe COVID-19-associated encephalopathies including ADEM or acute necrotizing encephalopathies and poly(radiculo)neuropathies as GBS or other acute neuropathies (multifocal demyelinating or small fiber polyneuropathy) have also been described in several case reports with an estimated prevalence of 0.1–1% in western countries within 6 weeks of confirmed infection (Mahapure et al. 2021; Parsons et al. 2020; Shahali et al. 2021; Toscano et al. 2020; Camdessanche et al. 2020; Hayley and Sun 2021; Dixon et al. 2020; Poyiadji et al. 2020; Oaklander et al. 2022). For some entities, a causative relationship to prior SARS-CoV-2 infection is less clear. For example, a large prospective observational study between January and May 2020 could not confirm a higher risk for GBS after SARS-CoV-2 infection (Luijten et al. 2021) and another study of 145.721 cases with COVID-19 showed that GBS had a low pooled prevalence (0.28% within the study cohort) compared to other neurological manifestations (Misra et al. 2021). Among the most common neurological complications in hospitalized patients with COVID-19 are cerebrovascular diseases which are likely associated with a pro-inflammatory hypercoagulable state in severe infection with elevated C-reactive protein, D-dimer, and ferritin (Lodigiani et al. 2020; Benussi et al. 2020). Patients presented with ischemic and hemorrhagic stroke or venous thrombosis. In a recent meta-analysis of 145,634 COVID-19 patients, of which 89% were hospitalized, every third patient presented with a neurological manifestation (Misra et al. 2021). A cross-sectional surveillance study in neurology, stroke, psychiatry, and intensive-care units in the UK in April 2020 included 125 patients of which 77 (62%) had a cerebrovascular event (74% ischemic, 12% intracerebral hemorrhage, and 1% vasculitis), while altered mental status was the second most common presentation (Varatharaj et al. 2020). In 267 cases that were followed-up during that time COVID-19 associated stroke was mostly observed in younger adults, delirium was common in patients over 60 years and encephalopathy mostly in patients under 60 years treated on intensive-care units (Ross Russell et al. 2021). Although less frequent, infections with SARS-CoV-2 have also been reported to result in movement disorders (Salari et al. 2021; Hull et al. 2021).

### Implications for patients with neurodegenerative disorders

Populations at risk for a deleterious outcome of COVID-19 have been identified early in the pandemic and encompass patients with higher age, male sex, obesity, diabetes, and other comorbidities (Williamson et al. 2020). Patients with neurodegenerative disease are at particular risk, being mostly of higher age and presenting with many relevant comorbidities (Kitani-Morii et al. 2021). Several studies have shown that a pre-existing neurodegenerative disease itself worsens SARS-CoV-2 infection outcomes. Data derived from meta-analyses and the UK Biobank have shown that dementia is an age-independent risk factor for a deleterious outcome of COVID-19 with increased disease severity and higher likelihood of death compared to non-demented control patients (Tahira et al. 2021; Liu et al. 2020a). This effect has been confirmed for the subgroup of patients with AD, while vascular dementia was not associated with a higher risk (Zhang et al. 2021b). The clinical presentation of COVID-19 infection in patients with concomitant dementia is often atypical with apathy and confusion or hyperactive delirium as leading symptoms, while cough, fever, or dyspnea are less frequent symptoms (Bianchetti et al. 2020; Harb et al. 2021; Mendes et al. 2021; Rebora et al. 2021; Hariyanto et al. 2021). In addition, delirium is an independent factor for worsening the outcome of SARS-CoV-2 including increased fatality (Rozzini et al. 2020; Garcez et al. 2020; Mendes et al. 2021). In most retrospective analyses, a higher case fatality rate has been reported in patients with PD. Particularly, PD patients with longer disease duration and higher age show a high mortality rate of 21–40% during SARS-CoV-2 infections in different cohorts in Italy, the US, and Germany and independent of age, sex and ethnic background (Antonini et al. 2020; Zhang et al. 2020; Scherbaum et al. 2021). On the other hand, some studies with smaller sample sizes recruiting patients in tertiary referral centers in Europe and Italy did not find an increased mortality rate in PD nor dementia compared to demographically matched controls (Huber et al. 2021; Fasano et al. 2020).

Data on other neurodegenerative diseases are less extensive. A study using the US veteran database and 699 patients with amyotrophic lateral sclerosis (ALS) found an increased risk of COVID-19 related death in this cohort (Galea et al. 2021). In a Scottish cohort of 1062 patients with motor neuron disease, 77.7% of whom were diagnosed with ALS, all-cause mortality remained unaffected in 2020, though specific mortality rates of individuals diagnosed with COVID-19 are not reported (Glasmacher et al. 2021). Interestingly, C9orf72 repeat expansions of intermediate length, a mutation often found in cases of familial ALS and frontotemporal dementia (FTD), have been associated with severe COVID-19 requiring mechanical ventilation (Zanella et al. 2021). Multiple sclerosis itself has not been associated with a severe course of disease, whereas increased disability and a progressive course of the disease, which is mainly mediated by neurodegenerative processes, have (Prosperini et al. 2022; Barzegar et al. 2021). Future prospective studies including all healthcare services may shed light on the actual risk of patients with neurodegenerative disorders.

Besides the direct effect of an infection with SARS-CoV-2 on patients with neurodegenerative diseases, the impact of the global COVID-19 pandemic on these patients due to disruptions in the medical care is equally alarming. Since onset of the global pandemic in late 2019, symptoms such as anxiety, depression, and sleep disturbances have been found to have increased in the general public (Thygesen et al. 2021; Bauerle et al. 2020). This is in line with cross-sectional data from more than 5000 patients with PD that revealed worsening of motor (43%) and especially non-motor (52%) symptoms since pandemic onset (Brown et al. 2020). Home confinement and other disease control measures aiming at reduction of contacts are likely to contribute to a diminished physical activity in PD patients (Leavy et al. 2021) and foster the discontinuation of medical treatments, such as multimodal complex treatment or levodopa/carbidopa intestinal gel (LCIG) set-ups (Richter et al. 2021; Wolff et al. 2022). Disruptions were furthermore observed to impact clinical trials, due to difficulties in recruitment, initiation and monitoring (Lorusso et al. 2020) which will impact the development of future therapeutic options. Although adaptions such as implementation of remote monitoring and remote study endpoints have been made, they need to be expanded in the future (Snider and Holtzman 2021).

### Potential long-term consequences of SARS-CoV-2 infections

**Fig. 1:**
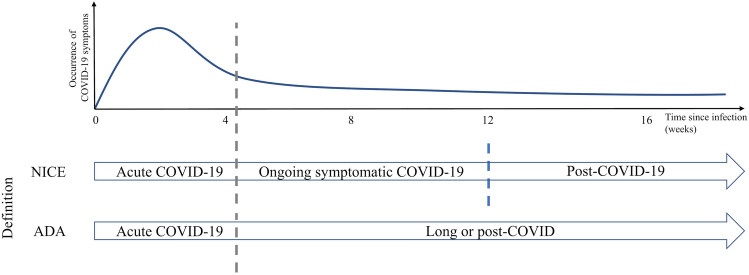
Terms used to describe persisting symptoms after SARS-CoV-2 infection. NICE, British National Institute for Health and Care Excellence; ADA, Americans with Disabilities Act

After acute infection with SARS-CoV-2, some of the associated symptoms can persist or additional symptoms can emerge. The British National Institute for Health and Care Excellence (NICE) distinguishes on one hand ongoing symptomatic COVID-19, defined as persistence of COVID-19-related signs and symptoms up to 12 weeks after infection, from the post-COVID-19 syndrome, defined as the persistence or occurrence of COVID-19-related symptoms 12 weeks after infection (Fig. 1) (NICE 2022). The term “long-COVID” refers to the entirety of symptoms after an acute SARS-CoV-2 infection independent of time and duration and can be considered a disability under the Americans with Disabilities Act (ADA) since July 2021 (Centers for Disease and Prevention 2021; HHS.gov 2021). Patients suffering from post-COVID-19 syndrome may present with a volatile cluster of symptoms in any system of the body. These may include typical COVID-19-related symptoms such as loss of smell or dyspnea, but also include neurological symptoms, such as cognitive impairment, dizziness, and delirium (NICE 2022). Longitudinal assessment of 9751 patients with COVID-19 found the persistence of COVID-19-related symptoms in 72.5% of the cases after discharge (follow-up range from 29 to 234 days), most prominently shortness of breath or dyspnea (36.0%), and fatigue or exhaustion (40.0%) (Nasserie et al. 2021; Lara et al. 2020). Particularly, the persistence of fatigue or muscle weakness correlated with the need of supplemental oxygen or the need of invasive and non-invasive breathing assistance in hospitalized COVID-19 patients 6 months after discharge (Huang et al. 2021). Both, the pandemic itself, and a previous infection with SARS-CoV-2 have been linked to an increase in distress and depression in the general public (Ramiz et al. 2021; Nasserie et al. 2021). In particular, patients with pre-existing neurodegenerative or chronic neurological diseases are at special risk for this development. Patients with dementia, AD, PD, and multiple sclerosis have been found to suffer from worsening of pre-existing symptoms and de-novo development of neuropsychiatric symptoms, e.g., anxiety and cognitive decline (Lara et al. 2020; Boutoleau-Bretonniere et al. 2020; Wei et al. 2022; Salari et al. 2020; Shalash et al. 2020; Wolff et al. 2022; Haji Akhoundi et al. 2020). This long-lasting impact of an infection with SARS-CoV-2 has resulted in the need for an implementation of a medical care structure for these patients (Gemelli Against 2020).

The association of neuropsychiatric symptoms with SARS-CoV-2 infection raises the question about the potential long-term impact of the virus on the CNS, its molecular basis, and the potential risk of neuronal damage associated with subsequent development of neurodegenerative diseases. During SARS-CoV infection, there is evidence of scattered degeneration of neurons as a sign of neuronal hypoxia/ischemia (Gu et al. 2005). Sepsis itself has long-term consequences on the brain often associated with delirium and even cognitive decline later in life. Several mechanisms have been discussed to cause neuronal damage during sepsis. First, decreased blood flow caused by reduction in peripheral vascular tone and diminished cardiac output alters brain circulation. Altered microcirculation of the brain, possibly also through an impaired autoregulation caused by increased CO_2_ levels, leads to detachment of pericytes from the basal lamina and increased permeability of the BBB (Nishioku et al. 2009; Barichello et al. 2021). Sepsis triggers the peripheral innate immune system with an increased secretion of TNF-alpha and interleukins causing endothelial cell damage, disintegration of intracellular junctions, and increased permeability of the BBB. Invasion of T-lymphocytes and macrophages via the BBB activates microglia and astrocytes leading to cerebral cytokine, chemokine, and nitric oxide release, which causes neuronal dysfunction and ultimately cell death (Heneka et al. 2020; Widmann and Heneka 2014). A well-established indicator for neuronal damage is the increase of blood or CSF levels of neurofilament light chain (NfL), which is a structural protein and part of the cytoskeleton of long axons. An increase in neurofilament levels has been related to unspecific neuronal damage that can be caused by neuroinflammation, neuronal ischemia, or neurodegeneration (Khalil et al. 2018). Several studies assessed neurofilament levels in SARS-CoV-2 patients. CSF and blood NfL levels were elevated in patients with severe disease and concomitant neurological symptoms (Virhammar et al. 2021; Paterson et al. 2021). Another study evaluated blood NfL levels of 47 SARS-CoV-2 patients at admittance to hospital and showed that a higher baseline NfL level is a negative predictor of survival (Aamodt et al. 2021). Blood NfL was increased in the acute phase of 48 severe and 28 moderate SARS-CoV-2 cases, while after a 6-month follow-up NfL was normalized (Kanberg et al. 2021). To predict the extent of neuronal damage in a cohort of 142 hospitalized patients with COVID-19, Prudencio et al. measured serum NfL at time of hospitalization and at multiple follow-ups (Prudencio et al. 2021). Higher baseline NfL levels indicated worse clinical outcome, while over time NfL levels stayed constant or fluctuated, without a clear correlation to clinical symptoms. Although neurofilaments are an indicator of neuronal damage, it is not yet clear if their elevation indicates a risk for long-term neurodegeneration. To further support neuronal loss, diminished gray matter within the limbic cortical areas with direct neuronal connectivity to the primary olfactory system has been recently described in longitudinal brain imaging after COVID-19 infection (Douaud et al. 2021).

**Fig. 2:**
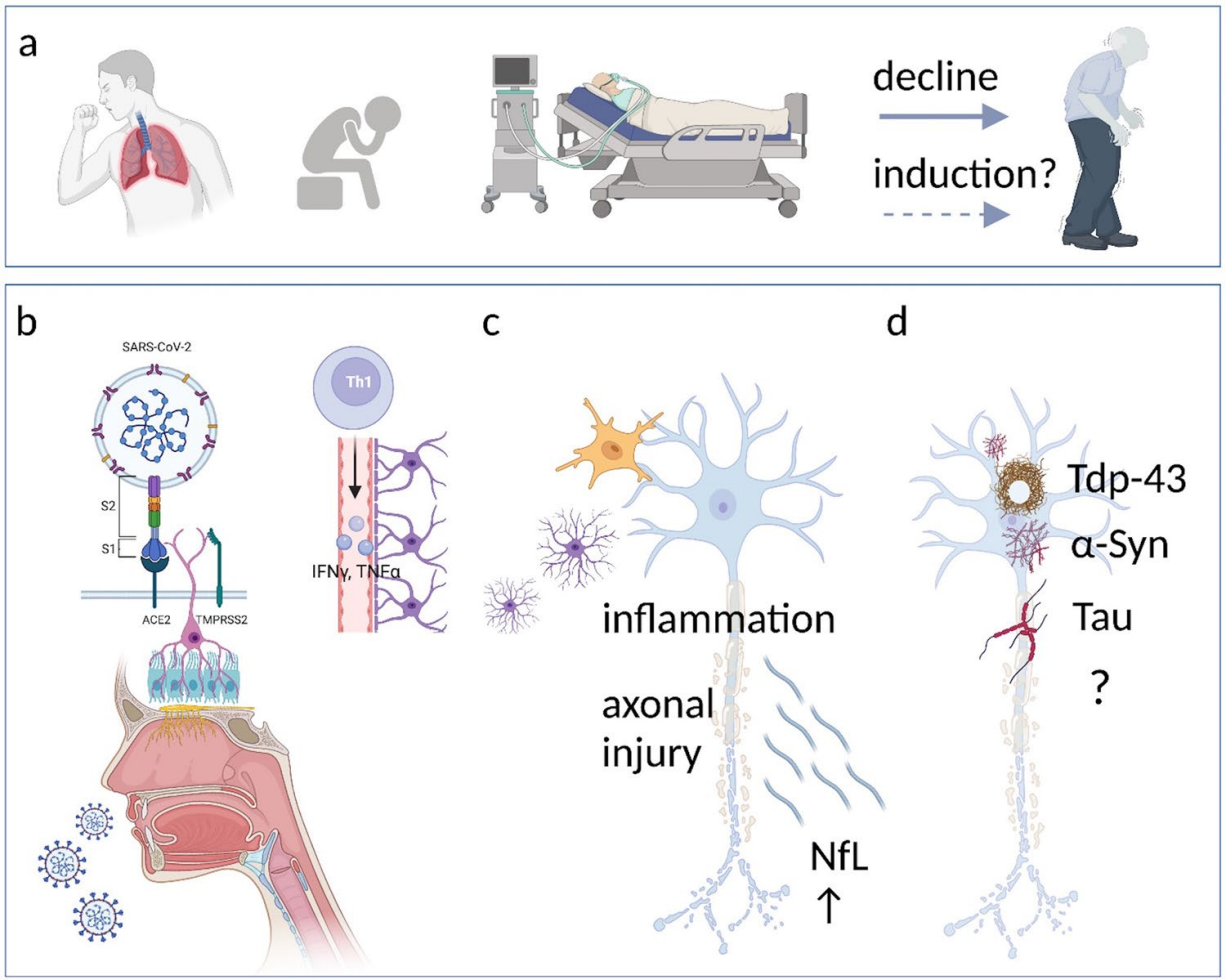
SARS-CoV-2 and neurodegenerative diseases. **a** Clinical presentation: SARS-CoV-2 infection leads to neurological symptoms and disorders (e.g., loss of smell, altered mental status, and stroke), severe sepsis, or worsening of neurodegenerative diseases. Whether SARS-CoV-2 induces neurodegenerative disorders is unknown. **b**–**d** Cellular and molecular mechanisms. **b** SARS-CoV-2 enters the brain via the nasal and oral mucosa and affects multiple brain regions, also those involved in neurodegenerative disorders. Systemic inflammation leads to dysfunction of the BBB and neuroinflammation. **c** Activation of microglia, astrocytes, and pro-inflammatory cytokines in the brain may trigger neuronal damage and release of NfL. **d** Neuronal damage by SARS-CoV-2 could be a potential risk for the development of neurodegeneration associated with proteinopathy, such as PD, AD and ALS. *PD* Parkinson’s disease, *AD* Alzheimer’s Disease, *ALS* amyotrophic lateral sclerosis, *NfL* neurofilament light chain, *Tdp-43* TAR DNA-binding protein 43, *a-Syn* alpha-synuclein, *Tau* Protein Tau (Created with https://biorender.com/)

Further evidence of a potential risk for the development of neurodegenerative diseases comes from case reports describing patients with akinetic-rigid parkinsonism following infection with SARS-CoV-2. In addition to improvement of symptoms either under Levodopa/Carbidopa-treatment or spontaneously, all patients displayed abnormal imaging findings, such as decreased dopamine transporter density in dopamine transporter scan or decreased dopamine uptake in ^18^F-DOPA PET (Cohen et al. 2020; Faber et al. 2020; Mendez-Guerrero et al. 2020). It remains uncertain, if these patients developed the clinical findings de-novo or if a subclinical PD emerged after worsening through severe COVID-19. Causal links of viral infections to neurodegenerative diseases have been drawn since the influenza pandemic in 1918, which is assumed to have caused numerous cases of postencephalitic parkinsonism (Kim et al. 2015; Hoffman and Vilensky 2017; Bigman and Bobrin 2018). This theory was further fueled by the fact that the substantia nigra represents a potential target for neurovirulent influenza A viruses (Takahashi et al. 1995). This is also true for coronaviruses, which have been shown to accumulate in the nucleus subthalamicus and the substantia nigra after infection in mice (Fishman et al. 1985). Furthermore, elevated titers of antibodies directed against coronaviruses have been found in patients with PD (Fazzini et al. 1992). ACE2 has not only been found to be expressed by midbrain dopaminergic neurons, but dopaminergic neurons were also permissive for an infection with SARS-CoV-2 in-vitro (Yang et al. 2020). Further evidence for the involvement of the midbrain during COVID-19 was added by the fact that post-mortem analysis of patients with COVID-19 revealed activated microglia and cytotoxic T-lymphocytes in the brainstem and cerebellum (Matschke et al. 2020). By infiltration of viruses to the CNS, neuroinflammation can be triggered, resulting in a disruption of the BBB, microglia activation, and clustering of microglia around (dopaminergic) neurons, as well as pro-inflammatory cytokine release. This is thought to result in a vicious circle of self-driven neuroinflammation, eventually resulting in neurodegeneration (Limphaibool et al. 2019). In PD, there is strong evidence suggesting alpha-synuclein pathology propagates from the olfactory bulb and/or the gut to the brain, which is associated with early non-motor symptoms such as olfactory disfunction and obstipation (Braak et al. 2003; Haehner et al. 2011). Olfactory dysfunction, in particular loss of smell, is also a common symptom of COVID-19. ACE2 and TMPRSS2 have been shown to be expressed in human olfactory mucosa and therefore pose a possible entry site of SARS-CoV-2 to the brain, which is shared by alpha-synuclein pathology (Fig. 2; Brann et al. 2020). Additionally, viral infections were found to increase alpha-synuclein expression levels in rodents and to induce the formation of cytotoxic alpha-synuclein aggregates (Beatman et al. 2015; Bantle et al. 2019). Whether and in what way these findings might contribute to the development of PD is unknown. Furthermore, SARS-CoV-2 has been shown to also influence other proteins involved in neurodegenerative diseases such as AD: the expression of SARS-CoV-2 spike protein S has been linked to an increased spreading of Tau and cytosolic prions in-vitro and further prompted the aggregation of these proteins (Liu et al. 2021). A shift in localization of Tau from axons to the soma and hyperphosphorylation of Tau, both hallmarks of early stages of tauopathy, have been observed in SARS-CoV-2-infected neurons, resulting in cell death in 3D human brain organoids (Ramani et al. 2020). In Amyotrophic lateral sclerosis (ALS), the main pathological protein found aggregated in the cytoplasm of neurons leading to progressive degeneration of motor neurons is TAR DNA-binding protein 43 (TDP-43). In 10% of ALS cases, a known gene mutation underlies the disease, while in the majority of sporadic ALS patients, a multi-hit hypothesis of internal and external factors including environmental risk factors has been considered (Brown and Al-Chalabi 2017). A viral infection of specific neuronal populations such as motor neurons might lower the threshold for developing TDP-43 pathology as proposed in the multistep hypothesis for ALS (Chio et al. 2018). In summary, there is growing evidence of COVID-19 associated neuropathological effects and SARS-CoV-2 may be impacting many pathways involved in the pathogenesis of neurodegenerative diseases. Whether this is sufficient to induce neurodegeneration and whether the human nervous system is able to counteract and regenerate is subject of ongoing research. Longitudinal data will be needed to evaluate the relevance of this impact on patients diagnosed with COVID-19.

### Open questions

This short review summarizes molecular and clinical evidence of CNS affection by the SARS-CoV-2 virus with particular reference to neurodegenerative disorders. Many questions, however, prevail. Although it is known that COVID-19 can cause neuronal damage as indicated by increased neurofilament levels, it is unresolved whether the CNS is able to recover completely or only partially from this damage and what the delay of such a recovery might be. Even less clear is whether such damage to the CNS triggers neurodegenerative diseases and with which latency the (clinical) onset of such diseases might be expected. Which genetic predispositions or environmental factors could modulate these outcomes? Are all viral strains/variants of concern equally dangerous in this regard? We also do not know whether long-COVID or post-COVID-19 syndrome predisposes to such potential long-term consequences. We know that vaccination against SARS-CoV-2 is a powerful means to prevent a severe course of disease in the general public (Thompson et al. 2021; Lin et al. 2022; Rosenberg et al. 2022; Hippisley-Cox et al. 2021). Data on the safety and effectiveness of the available vaccines in the vulnerable population of patients with neurodegenerative diseases are lacking and urgently needed (Shi et al. 2021). It is also not clear to date whether vaccination also prevents potential neurodegeneration as sequelae of COVID-19 and what the role of different vaccines may be. Only longitudinal studies with large cohorts and appropriate controls will be able to solve these questions in the future (Horn et al. 2021).
